# Leber's Hereditary Optic Neuropathy-Gene Therapy: From Benchtop to Bedside

**DOI:** 10.1155/2011/179412

**Published:** 2010-12-26

**Authors:** Rajeshwari D. Koilkonda, John Guy

**Affiliations:** ^1^Bascom Palmer Eye Institute, Miller School of Medicine, University of Miami, Miami, FL 33136, USA; ^2^McKnight Vision Research Center, Miller School of Medicine, University of Miami, Miami, FL 33136, USA

## Abstract

Leber's hereditary optic neuropathy (LHON) is a maternally transmitted disorder caused by point mutations in mitochondrial DNA (mtDNA). Most cases are due to mutations in genes encoding subunits of the NADH-ubiquinone oxidoreductase that is Complex I of the electron transport chain (ETC). These mutations are located at nucleotide positions 3460, 11778, or 14484 in the mitochondrial genome. The disease is characterized by apoplectic, bilateral, and severe visual loss. While the mutated mtDNA impairs generation of ATP by all mitochondria, there is only a selective loss of retinal ganglion cells and degeneration of optic nerve axons. Thus, blindness is typically permanent. Half of the men and 10% of females who harbor the pathogenic mtDNA mutation actually develop the phenotype. This incomplete penetrance and gender bias is not fully understood. Additional mitochondrial and/or nuclear genetic factors may modulate the phenotypic expression of LHON. In a population-based study, the mtDNA background of haplogroup J was associated with an inverse relationship of low-ATP generation and increased production of reactive oxygen species (ROS). Effective therapy for LHON has been elusive. In this paper, we describe the findings of pertinent published studies and discuss the controversies of potential strategies to ameliorate the disease.

## 1. Introduction

Leber's hereditary optic neuropathy (LHON) refers to a rare, neurodegenerative maternally inherited, mitochondrial genetic disease. The clinical features of LHON were first described by the German ophthalmologist Theodor Leber, in 1871 [1]. LHON is characterized by sudden painless loss of central vision. In the acute stages, the optic nerve head is swollen but later becomes atrophic [2]. Generally, visual loss is sequential with involvement of the second eye occurring weeks to months after the first [3, 4]. The mode of inheritance of LHON was thought to be X-linked, until the first report by Erickson in 1972, that described a non-Mendelian pattern of inheritance involving mitochondria [5]. In 1988, Wallace and his group reported the first mitochondrial DNA point mutation associated with LHON. It was the G to A transition at nucleotide 11778 in the *ND4* gene (NADH-ubiquinone oxidoreductase, subunit 4) of Complex I of the ETC that resulted in a substitution of histidine for arginine at amino acid position 340.

The disease shows variable penetrance with a male preponderance of 86% [6], and LHON is transmitted solely through females. The expression of LHON is heterogeneous. In some pedigrees, cardiac and neurological abnormalities have been documented along with the characteristic optic atrophy [7–13], loss of the retinal nerve fiber layer, and ganglion cells [14]. Fibers of the papillomacular bundle are highly sensitive to the degenerative process [15].

## 2. Epidemiology

There are few epidemiological studies of LHON. One of the largest was a population-based study done in the North-East of England. It reported a minimum point prevalence of visual failure to be 1 in 31,000. The minimum point prevalence was 1 in 8500 carriers of mitochondrial DNA mutations to be at risk of visual failure [16]. A similar prevalence was reported by two recent studies, one in the Netherlands of 1 in 39,000 and the other in Finland of 1 in 50,000, respectively, [17]. Analysis of Australian pedigrees showed approximately 0.42–2% of LHON-induced visual loss with a variability of prevalence among men (range: 7–58%) and women (range: 0–15%) [18]. Another study on a Finnish population reported the incidence based on the type of mutation. The prevalence of families with the 11778 mutation was 39% among men and 14% in women. In families harboring the 3460 mutation, the disease expressed in 32% of men and 15% of women [17].

## 3. Clinical Manifestations of LHON

**Figure 1:**
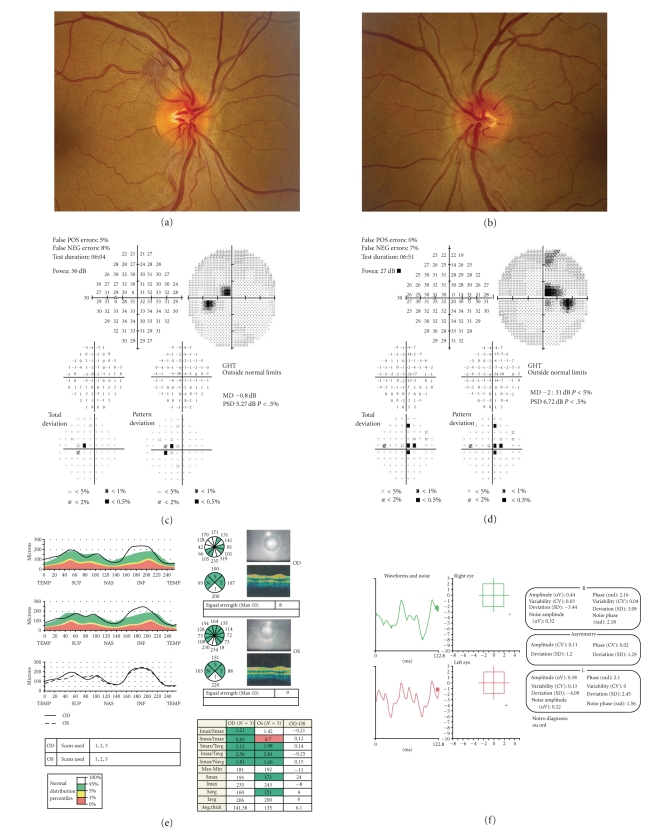
Fundus photographs of a patient with acute LHON revealed swelling of the right (a) and left (b) optic nerve heads. The arrow indicates the characteristic peripapillary telangiectasia. Automated visual fields showed central scotomas in the left (c) and right (d) eyes. OCT confirmed and quantitated the swelling of the retinal nerve fiber layer (e). Pattern electroretinograms illustrated a decline in ganglion cell function occurred during the acute stages of LHON and before structural evidence of RGC loss (f).

Patients with LHON typically present with acute or subacute, sudden, painless, central vision loss leading to central scotoma and dyschromatopsia [14]. Ophthalmoscopic examination reveals peripapillary telangiectasia, microangiopathy, swelling of the optic nerve head, and vascular tortuosity (Figure 1) [3]. This later progresses to optic atrophy. Visual dysfunction usually starts at 18 to 30 years of age. However, it may range from 3 to 80 years, with a mean age of onset being 25 to 26 years in men and 27 to 29 years in women [19]. In most cases, visual deterioration is rapid and extreme, with Snellen visual acuities of 20/200 or even worse in each eye. In a few cases, visual loss is slow and insidious over a period of 2 years, with a mean progression time of 3.7 months.

The probability of spontaneous recovery among LHON patients varies depending on the causative mutation. The highest recovery rate occurs in patients with the 14484/ND6 mutation (37% in a period of 16 months). Patients possessing the 11778/ND1 mutation have the poorest recovery rate (4%) [6, 20, 21].

LHON can also be associated with minor neurological abnormalities defined as Leber's “plus.” A study by Nikoskelainen et al. reported that 59% of their LHON patients harboring any of the three primary mtDNA mutations had neurological abnormalities [10]. Clinical manifestations included postural tremor, motor disorder, Parkinsonism with dystonia, peripheral neuropathy, multiple sclerosis-like syndrome, cerebellar ataxia, anarthria, dystonia, spasticity, or mild encephalopathy [10, 22–25].

Previous reports on the influence of environmental factors such as cigarette smoking and alcohol consumption on LHON pathology had somewhat contrasting results [26, 27]. A recent large study by Kirkman and his group showed no statistically significant association of smoking to LHON. They reported that carriers with heavy smoking habits were greatly susceptible to developing the disease, but the effects of alcohol were not statistically significant [28]. A single case report demonstrated the influence of malnutrition along with tobacco abuse as risk factors for LHON [29].

## 4. Genetics

Three-point mutations in the mtDNA respiratory chain Complex I subunit genes: m.3460G>A/ND1 [30, 31], m.11778G>A/ND4 [6], and m.14484 T>C/ND6 [32] are associated with LHON worldwide. They constitute approximately 95% of the LHON pedigrees belonging to patients of northern European descent [33]. These three mtDNA mutations are considered to be the primary pathogenic mutations, as they alter evolutionarily conserved amino acids, and they are absent in the control individuals [30–32, 34]. In a small number of LHON cases, secondary mtDNA mutations that do not change evolutionarily conserved amino acids may be causative. In addition, a synergistic mechanism has been proposed whereby the secondary mutations along with the primary mutations increase the severity of LHON [35]. Since secondary mutations are also detected in unaffected control individuals, they may simply represent polymorphisms of the mitochondrial genome. The human mitochondrial genome database (MITOMAP:www.mitomap.org) lists most of the variants of mtDNA. A comprehensive list of all the mtDNA mutations associated with LHON is shown in Table 1. 

Since all individuals with the pathogenic mtDNA mutations do not develop visual loss, the incomplete penetrance of LHON may be due to other genetic (nuclear or mitochondrial) or epigenetic factors. The mtDNA haplogroup is another major genetic determinant for LHON [36]. The mtDNA haplogroups include the nonsynonymous variants in Complex I and III subunit genes. European haplogroup J is preferably associated with the 11778/ND4 (32%) and 14484/ND6 (70–75%) pathogenic mutations. This haplotype increases the risk of visual loss [37–39]. The haplogroup J is further classified based on the amino acid changes in the cytochrome b gene [40]. There is an association of haplogroup J1 with the 14484 mutation and J1c and J2b with the 11778 mutation, thereby indicating the influence of specific combinations of amino acid changes influencing the mitochondrial respiratory chain Complexes I and III [40]. In addition, the 14484 LHON mutation showed low penetrance when present in the haplogroup H mtDNA background [41]. However, the distribution of the 3460 mutation was random among the haplotypes [37–39]. *In vitro* studies on cybrids with mitochondria that carry the 11778 on the haplogroup J background put onto the neutral nuclear background of osteosarcoma cells had lower oxygen consumption and delayed mitosis as compared to a nonhaplo J genotype [42]. However, another study reported no detectable differences in respiratory function between cybrids belonging to European haplogroups X, H, T, or J [43]. Recently investigation of the effect of mtDNA haplogroups on the assembly of oxidative phosphorylation (OXPHOS) complexes showed a differentially delayed assembly rate of respiratory chain Complexes I, III, and IV amongst mutants belonging to different mtDNA haplogroups. This indicates that specific mtDNA polymorphisms may modify the pathogenic potential of LHON mutations by affecting the overall assembly kinetics of OXPHOS complexes [44]. The influence of exposure to n-hexane neurotoxic metabolite 2,5-hexanedione (2,5-HD) on cell viability and mitochondrial function of different cell models (cybrids and fibroblasts) carrying the LHON mutations on different mtDNA haplogroups was studied. Cell death induced by 2,5-HD was greatly increased in LHON cells carrying the 11778/ND4 or the 14484/ND6 mutation on the haplogroup J background. On the other hand, the 11778/ND4 mutation in association with haplogroups U and H significantly improved cell survival [45]. Hence, the mtDNA haplotypes might act in association with the pathogenic mtDNA mutations to somehow modulate the phenotypic expression of LHON.

## 5. Heteroplasmy and Incomplete Penetrance

LHON exhibits incomplete penetrance with a male predominance. Approximately half of the men and 10% of females harboring one of the three pathogenic mtDNA mutations develop visual loss. This suggests that additional genetic factors and/or environmental factors modulate the phenotypic expression of LHON. The male preponderance in the disease manifestation could be also due to other anatomical, hormonal, or physiological factors [46].

Generally, cells contain 100–10,000 mitochondria, and each organelle harbors 2–10 mtDNA molecules. The copy number of mtDNA is therefore very high and shows heterogeneous distribution in different tissues based on the energy requirements [47]. Homoplasmy is defined as all the mitochondria of the cell possessing either wild-type or mutant mtDNA. When there is a mixture of wild-type and mutant mtDNA, it is called heteroplasmy. In the majority of LHON patients and family members, the pathogenic mtDNA mutation is homoplasmic. Still, 14% of the LHON members have the mutation in heteroplasmic condition [48]. Clinically, there are no differences among the affected homoplasmic individuals from heteroplasmic patients [35]. 

Jacobi et al. reported variable prevalence of heteroplasmy based on the type of mutation possessed in 167 genealogically unrelated LHON families. Individuals with 11778/ND4, 3460/ND1, and 14484/ND6 mutations showed levels of heteroplasmy equal to 5.6%, 40% and 36.4%, respectively, [49]. However, a study analysing four large Thai LHON pedigrees showed a prevalence of 37% of heteroplasmic 11778/ND4 mtDNA [50, 51]. In heteroplasmic families, the level of heteroplasmy can vary extensively between generations and also between offspring in the same family due to a genetic bottle neck effect of mitochondrial distribution occurring in the early stages of oocyte formation [48, 52–55]. 

In addition, there have been controversial reports on the distribution of mutant mtDNA in different tissues. Yen et al. compared the mutant mtDNA from the leukocytes and hair follicles in an LHON proband carrying the 11778/ND4 mutation and observed mtDNA heteroplasmy in the hair follicle cells, but not in blood cells. This finding indicated the tissue variability in distribution of the wild-type to mutant mtDNA [56]. However, another report demonstrated comparable levels of mtDNA heteroplasmy in the blood, hair, and urinary tract epithelia of LHON patients carrying the 11778/ND4 mutation [57].

In some heteroplasmic LHON families, an increase in the proportion of the mutant mtDNA in successive generations has been observed [48, 54]. This finding suggests a positive selection pressure. However, Puomila et al. quantified the level of heteroplasmy of the mtDNA mutations 11778/ND4 and 3460/ND1 in blood samples over a period of 4–12 years from nine members of four heteroplasmic LHON families. No major shift in heteroplasmy was demonstrated, thus no selection of either mtDNA genotypes. They proposed that the segregation of the wild-type mtDNAs and those carrying LHON mutations is a stochastic process governed by random genetic drift. In this respect, LHON mutations seem to behave like neutral polymorphisms [58]. These observations indicate that the role of selection is questionable.

The risk of visual failure in LHON increases as the threshold of heteroplasmy of primary pathogenic mtDNA mutations is increased to approximately 75%–80% [59]. The effect of heteroplasmy on phenotypic expression does not appear to be related to gender [60]. Howell et al. demonstrated in autopsied specimens of a woman with the 11778/ND4 mutation that the mutant mtDNA level was higher in the optic nerves (95%) and retina (100%) compared to circulating blood leukocytes (33%) [54]. This finding suggests that the susceptibility of certain tissues is due to their higher threshold of mutant to wild-type mtDNA. However, in a family of LHON, one of the two brothers with 98% mutant mtDNA lost vision, while his brother who had 100% mutant mtDNA was asymptomatic. Still, their ocular levels of mutated mtDNA were not evaluated. 

The proposed risk for disease expression in homoplasmic families is only 30–50% in males and 5%–15% in females [35]. Chinnery et al. studied 17 independent LHON pedigrees to determine the risk of transmission of LHON in heteroplasmic families. He reported that mothers with 80% or less mutant mtDNA (measured in blood leuckocytes) were less likely to have clinically affected sons than mothers with 100% mutant mtDNA [61].

## 6. Mechanisms of Cell Death in LHON

Despite the presence of the mtDNA mutation in all retinal cells, it is predominantly RGCs of the papillomacular bundle region of the retina and their axons in the optic nerve that undergo degeneration in LHON. Why the disease spares other cell types such as the photoreceptors and the retinal pigment epithelium is unclear but may in part be due to the unique energy demands of RGCs, with their long axons and transition from unmyelinated to myelinated fibers in the retrobulbar nerve. Mitochondrial dysfunction from energy depletion has been proposed to disrupt axonal transport [61, 62]. Axonal transport is driven by the motor proteins kinesin and dyenin, both of which require large amounts of ATP for this function [61]. Therefore, proteins synthesized in the RGC cytoplasm as well as the mitochondria themselves that do not move down the axon towards the brain may contribute to the visual loss and degeneration of LHON.

As most LHON mutations involve the NADH-ubiquinone oxidoreductase, a decrease in Complex I activity resulting in apoptotic cell death is paramount [63]. LHON cybrids grown in galactose media, as the sole carbon source, force the cells to rely on oxidative phosphorylation rather than glycolysis to generate ATP. Under such restrictive conditions, LHON cells with mutated mitochondrial DNA undergo apoptotic cell death in a calcium [Ca(2+)-]dependent [64] and caspase-independent pathway [65, 66]. In addition, cytochrome c along with the apoptosis inducing factor (AIF) and endonuclease G (Endo G) are released from the mitochondria into the cytosol. Control cells with normal mitochondrial DNA remained unaffected by this restrictive media [65]. Cells harboring the 3460 and 14484 mtDNA mutations in the same nuclear background were comparitively more sensitive to apoptotic death than those harboring the 11778 mtDNA mutation. Battisti et al. treated the peripheral blood lymphocytes of LHON patients and controls with 2-deoxy-D-ribose and found a higher apoptotic rate in cells of LHON patients in comparison to controls, thus indicating mitochondrial involvement in this susceptibility [67].

In addition, mechanisms relating to increased oxidative stress have been proposed in LHON pathophysiology. Studies on the osteosarcoma-derived cybrids made from the mitochondria of LHON patients, carrying the 11778/ND4, 3460/ND1, or the 14484/ND6 mtDNA mutations, showed an excitotoxic mechanism of impaired glutamate transport. Defective activity of the excitatory amino acid transporter 1 (EAAT1) led to oxidative stress and increased mitochondrial ROS within RGCs. This in turn contributed to the apoptotic pathway of cell death of RGCs, loss of axons, and optic nerve atrophy [68].

**Figure 2:**
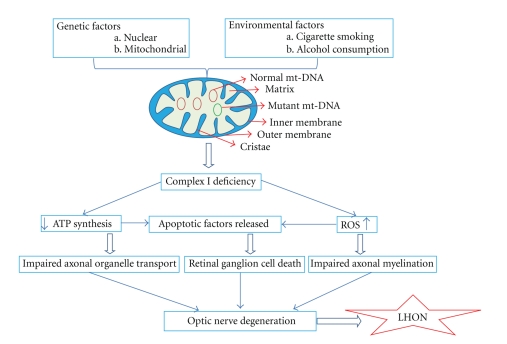
A schematic diagram illustrates the interaction of Complex I dysfunction, decreased ATP production, increased ROS, and apoptosis that culminate in the optic nerve degeneration of LHON and LHON cellular and animal models. ATP—adenosine triphosphate; ROS—reactive oxygen species.

In a study of oxidative stress of a cell line previously thought to be of RGC lineage (RGC-5), it was found that endogenous levels of superoxide anion were significantly lower than that found in neurons of the rat brain. Increases in ROS caused by mtDNA mutations that trigger the apoptotic cascade in ganglion cells of the retina may be better tolerated by neurons of the brain [69]. That mitochondrial DNA mutations result in Fas-induced apoptosis were demonstrated in osteosarcoma-derived cybrid cells carrying the 11778/ND4 or 3460/ND1 mutations. Control cells with the same mitochondrial halogroup J, but without the pathogenic G11778A mutation, were not sensitive compared to other controls. This finding indicates the pathogenicity of the LHON mutations [70].  Figure 2 shows potential pathways implicated in the optic nerve degeneration of LHON, as deduced from cellular and animal models.

## 7. Current Therapies

Management of LHON has been supportive, primarily by the use of low-vision aids. Current therapies are inadequate, but they deserve mention. The mainstay of treatment includes pharmaceutical compounds that are believed to restore electron flow or increase antioxidant defenses. One of these agents is idebenone, a short chain derivative of coenzyme Q_10_ (CoQ_10_) [71–73]. Mashima et al. used idebenone combined with vitamin B2 and vitamin C, to “stimulate ATP formation” in LHON patients. Treatment for at least one year hastened the recovery process. Visual improvement that was defined as being greater or equal to 0.3 logMAR occurred within 17.6 months of treatment relative to 34.4 months without it [73]. In another report of idebenone and vitamin B12 therapy, a North African LHON patient harboring a homoplasmic 14484/ND4 mtDNA mutation recovered vision. Serum lactate levels normalized over a period of 3.5 months [72]. In contrast, two other patients who were treated with idebenone and multivitamins failed to improve [74]. The effectiveness of idebenone therapy for LHON is currently the subject of a controlled double-masked randomized study in Europe and Canada. While the results of a recent press release were favorable, details have not yet been published.

The clinical phase of the patient at which time therapy is initiated might determine treatment outcome. As a prophylactic measure to prevent vision loss, a topically applied agent, brimonidine purite 0.15% (Alphagan), with potentially antiapoptotic properties was administered to the as yet unaffected eyes of LHON patients. Unfortunately, this therapy proved unsuccessful in preventing them from undergoing visual loss. Thus, the study was terminated after enrollment of only 8 patients [75]. The search for an effective treatment continues.

## 8. Animal Models

The genetics of LHON have steadily accumulated for more than two decades. However, the pathogenic mechanisms leading to the apoplectic visual failure with subsequent retinal ganglion cell and optic nerve degeneration that could lead to the development of an effective treatment strategy are poorly understood. This is in large part due to the lack of bona fide animal models for LHON. The deficiency of animal models is also a general problem for most mitochondrial diseases, where the complete deletion of any subunit of the respiratory chain often results in a lethal phenotype [76]. Still, using different approaches, a few animal models resembling LHON have been generated in recent years. The first animal model for LHON was made by Zhang and his group by administering rotenone, an irreversible Complex I inhibitor, to mice. Histologic analysis showed thinning of the RGC layer, by 43%, one day after the rotenone injections [77]. 

Next, Qi et al. used a genetic approach to knockdown Complex I activity. They designed ribozymes to degrade the mRNA encoding a critical nuclear-encoded subunit gene of Complex I (NDUFA1). It markedly reduced Complex I activity in murine cells. Using the AAV vector as a vehicle to deliver the ribozymes into the mouse vitreous cavity, the authors found loss of RGCs and axons that resembled the histopathology of LHON [78]. This model system also implicated oxidative stress in the pathogenesis of the degenerative process.

As further evidence for involvement of ROS in optic nerve degeneration, intraocular injections of AAV-expressing hammerhead ribozymes designed to degrade mitochondrial superoxide dismutase (*SOD2*) mRNA induced further loss of axons and myelin in the optic nerve and ganglion cells of the retina, the very hallmarks of LHON histopathology [79]. RGC and axonal loss were ameliorated by intraocular injections of an AAV overexpressing *SOD2* into eyes that also had received the NDUFA1 ribozymes [80]. Later, Qi and associates proposed augmenting mitochondrial antioxidative defensive mechanisms to rescue cybrid cells with the G11778A mutation in mtDNA from galactose-induced apoptotic cell death by infecting them with AAV-*SOD2*. The control cells were treated with AAV-GFP (green fluorescent protein). Within 2 and 3 days of growth in galactose media, LHON cell survival increased by 25% and 89%, respectively, [81]. The ROS superoxide anion has recently been shown to mediate apoptosis in RGCs [82]. Dismutation of the superoxide anion by SOD suppressed RGC apoptosis. Taken together, these findings suggest that antioxidant genes may offer a therapeutic strategy directed at the pathophysiologic mechanisms of LHON.

Still, it was unclear whether such findings in those mouse models are truly representative of events in LHON patients. The ribozyme and rotenone animal models illustrate the pathogenic effects of severe loss of Complex I activity in the vertebrae visual system. However, the activity of the NADH-ubiquinone oxidoreductase is reduced only slightly in cells with the G11778A mtDNA. To generate a more representative animal model of LHON, Qi and associates constructed a mutant ND4 subunit gene that was designed to express the arginine-to-histidine substitution at amino acid 340 characteristic of the mutant human LHON ND4 protein [83]. Delivery of this construct with the AAV vector injected into the mouse vitreous cavity resulted in optic nerve head swelling. Several months later, the optic nerve became atrophic and ganglion cells of the retina were lost. Both optic nerve head swelling and visual loss are characteristics of acute LHON. They are followed by optic atrophy. Thus, the phenotype of the murine model and the human disease appear comparable.

Ultrastructural analysis of mutant ND4-injected eyes revealed disruption of mitochondrial cytoarchitecture, elevated reactive oxygen species that culminated in apoptosis of RGCs [83]. Mouse eyes injected with AAV containing the normal human ND4 showed no evidence of pathology whatsoever. Since the mutant and human ND4 constructs differed only in the arginine to histidine transition at amino acid 340 (mutant ND4), these studies affirm that the pathogenicity of this mutation is the cause of LHON. Ellouze et al. who later introduced the mutant human ND4 gene into rat eyes found that it caused RGC degeneration and a decline in visual performance [84]. An important difference between human LHON and rodent models is worth mentioning. Disease in rodents occurred even in the presence of endogenous mouse (or rat) ND4. In human LHON, wild-type ND4 is typically absent. That being the case, rescue of the LHON rodent model by the addition of more wild-type ND4 may not be possible.

## 9. Genetic Therapy and Future Directions

Curative treatments for mitochondrial disorders are currently lacking. However, extensive exciting research advances are being made. One of the most promising emerging technologies is “allotopic expression,” wherein a nuclear version of the mitochondrial gene is constructed by partially recoding the mtDNA gene in the nuclear genetic code. It was through allotopic expression of the mutant human ND4 subunit gene that an LHON-like phenotype was induced in rodent models as discussed in the previous section. Changing the ATA codon to ATG is necessary to achieve allotopic expression, since the ATA encodes for methionine in mitochondria, but isoleucine in the nucleus. In addition, the TGA codon that specifies tryptophan in mitochondria is a stop codon in the nucleus. Therefore, this codon must also be corrected for the full-length ND4 protein to be translated on cytoplasmic ribosomes. Protein import into mitochondria is then directed by the addition of a mitochondrial targeting sequence (MTS) to the amino terminus [85, 86]. Protein expression can then be monitored with an epitope tag appended to the carboxy terminus. Guy et al. were the first to use this approach with a human ND4 gene to rescue the defects of oxidative phosphorylation in G11778A LHON cells [86]. They constructed a synthetic ND4 subunit from overlapping 80 mer oligonucleotides. After packaging in an adenoassociated viral vector, it was used to transduce cells harboring 100% G11778A-mutated mtDNA. One of their constructs successfully increased ATP synthesis by threefold in LHON cell lines relative to controls treated with GFP or transduced with the same ND4 gene that had a different MTS and epitope tag that was not imported into the mitochondria [86].

**Figure 3:**
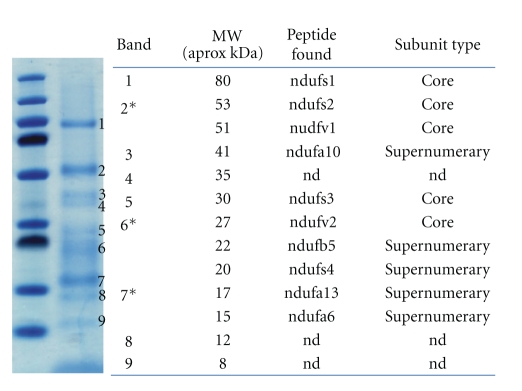
Electrophoresis of mitochondrial proteins isolated from the mouse brain following Complex I immunoprecipitation and counterstained by Coomassie blue are shown in the second lane. The first lane has the molecular weight standards. The table indicates the identity of bands submitted for mass spectroscopy to be subunits of Complex I. (*)—subunits identified within the same excised band. (nd)—not determined.

Last year, our group demonstrated that allotopic delivery of the normal human ND4 subunit gene into the vitreous cavity of the murine eye is safe. There was no difference in total RGC counts, measured as Thy1.2 positive cells, between these experimental eyes and controls injected with AAV-GFP. Moreover, the pattern and flash electroretinogram amplitudes after the injections remained unchanged from their baseline values before the injections. This important finding indicates that injection of the allotopic human ND4 did not compromise murine RGC function [87]. Using immunoprecipitation of the 45 subunit Complex I, we demonstrated that the FLAG-tagged human ND4 incorporated into the holoenzyme of infected murine retinal and optic nerve tissues. To validate the technique, we submitted nine bands pulled down by Complex I immunoprecipitation of murine mitochondria isolated from the optic nerve, brain, spinal cord, or retina for identification by mass spectroscopy. They were positively identified as subunits of the NADH-ubiquinone oxidoreductase (Figure 3). No other respiratory Complexes (II–V) were detected. Therefore, the FLAG-tagged human ND4 detected by this assay proves that it effectively integrated into the murine Complex I.

Prior evidence of cross complementation in dissimilar mammalian species was previously shown by Tsukihara and coworkers [88], where a bovine allotopic COX1 had integrated into the human cytochrome oxidase enzyme. They used blue-native electrophoresis to pull down the assembled COX holoenzyme. Using this technique, Figueroa-Martínez and coworkers [89] were unable to find integration of their construct that used a short COX6 MTS to direct import of ND6 tagged with hemagglutinin (HA), the only construct tested. Brookes and coworkers [90] used mass spectroscopy to identify more than 30 bands that were separated by 2D blue-native PAGE. Of these bands, a single 33 kilodalton (kDA) subunit of Complex I was identified [90]. In addition, the authors pulled down many proteins that were not respiratory complexes. The authors further go on to show that inhibition of mitochondrial protein synthesis of hydrophobic mtDNA-encoded proteins with chloramphenicol did not alter the assembly of respiratory complexes, as judged by blue-native electrophoresis. Therefore, unambiguous data showing that hydrophobic Complex I subunits are isolated by blue-native electrophoresis has yet to be demonstrated. 

Using electron microscopy as further evidence of allotopic import, ND4 labeled by immunogold decorated the interior of the organelle and it colocalized with MnSOD [83, 87]. The latter is a nuclear-encoded mitochondrial protein that too is imported into the organelle along with 85% of all mitochondrial proteins that are encoded by the nucleus. Had the ND4FLAG immunogold been stuck in the membrane pores as suggested by Oca-Cossio [91] it would have decorated the exterior of the organelle as shown by Gilkerson and colleagues [92] for the membrane protein porin. Thus, the allotopic ND4 protein did not get stuck in the mitochondrial import channels or induce cell death as suggested by Oca-Cossio and coworkers [87, 91]. Injections of wild-type human ND4 also had a small, but quantifiable, biological effect that was manifested by shortening of the pattern electroretinogram (PERG) latency. The implications of this finding are unclear. With the mouse and human ND4 being approximately 80% homologous and amino acid 340 being highly conserved, along with the fact that the latency of the mouse PERG is almost double that of humans, it is tempting to speculate that human ND4 integration into the mouse Complex I made the mouse RGC responses faster than that observed in control eyes. This may reflect an evolutionary adaptation of the human respiratory chain for the relatively fast RGC responses required in man. 

Controversies in allotopic expression based entirely on studies of cultured cells continue to be debated in the recent literature. However, they have shifted somewhat from the views of Oca-Cossio [91] that showed no colocalization of allotopic proteins (except ATP8) with a bona fide mitochondrial marker to those now showing colocalization suggestive of import, but lacking integration into functional respiratory complexes [89]. In 2002, Guy et al. showed that LHON cells expressing the same allotopic human ND4, but fused to a different mitochondrial targeting sequence (aldehyde dehydrogenase) or epitope tag (GFP) did import into the mitochondria [86]. Consistent with this finding, the latter construct did not rescue LHON cells from glucose-free galactose media-induced cell death or improve their ATP synthesis. 

In support of a paramount role for the MTS in directing mitochondrial trafficking, Superkova and coworkers [93] showed that the allotopic import of a mutant COX2 was dependent on the mitochondrial targeting sequence, but not the mitochondrial targeting 3′UTR. Still, the studies of Bonnet et al. [94] clearly demonstrated the benefits of the *COX10 *3′UTR when used in conjunction with the CIS acting elements of the *COX10* MTS. Relative to controls, the *COX10*-*ND4* or *COX10-ND4 *3′UTR constructs each increased G11778A LHON cell survival in galactose media and improved their ATP synthesis. Their findings support the earlier studies of Guy and coworkers' successful allotopic ND4 import into mitochondria. Clearly, the testing of constructs for allotopic expression that include the mitochondrial targeting sequence, protein, epitope tag, or 3′UTR is largely a trial-and-error endeavor [95]. Since cell culture studies can sometimes be misleading [96], confirmation in appropriate animal models is vital to demonstrating the safety and effectiveness of allotopic ND4 expression before it can be applied to LHON patients.

Using their MTS and 3′UTR model system, Ellouze and colleagues introduced the mutant human ND4 subunit gene harboring the G11778A mutation into rat eyes, by *in vivo* electroporation. This led to loss of vision and degeneration of almost half the RGCs, as previously described by Qi and coworkers [83]. By introducing a normal copy of the human ND4 gene, visual and RGC loss were averted [84]. Thus, the data accumulated to date provide overwhelming evidence that allotopic expression of a mutant ND4 causes RGC degeneration and a wild-type version does not. More importantly, they show that the wild-type ND4 can rescue an LHON animal model. Clearly, allotopic delivery of a normal ND4 is a promising approach in the quest for an effective remedy for LHON caused by mutated G11778A mtDNA.

For this to occur, an effective and safe delivery system is necessary for ND4 gene therapy. The single-stranded (ss) AAV2 used in allotopic mouse experiments has been proven safe in several phase I human ocular gene therapy trials [97]. Thus, the AAV vector has a proven track record in human clinical trials [98–101]. There has been extensive research on these viral vectors with much advancement, particularly in the areas of transduction efficiency, stability, tropism, and most importantly safety. The retinal layer exclusively affected in LHON can be targeted by optimizing the vector serotype (AAV2), and by choosing the route of vector administration (intravitreal injection). 

Newer generation vectors include the self-complementary (sc) AAV that contains both positive and negative complementary strands. Since second strand synthesis is believed to be the rate-limiting step for expression of single-stranded vectors, it is not surprising that scAAV vectors increase the speed and efficiency of transgene expression [102–104]. Other AAVs with mutations in the capsid proteins also increase the efficiency of transgene expression [105, 106]. They were designed to reduce cellular degradation of AAV, thus increasing cellular levels of AAV virions. By taking advantage of scAAV to deliver the allotopic ND4 into the mouse eye, our group doubled RGC expression relative to the single-stranded AAV that is the current standard vehicle for gene delivery. With scAAV, FLAG-tagged ND4 was seen in almost all murine RGCs (90%) [107]. Such newer generation vectors may be highly advantageous for LHON gene therapy. They can be used at lower doses, thus minimizing immunologic responses against the viral capsid that could prevent expression of ND4 and also with maximal efficiency [108]. This could have important implications for treatment of LHON patients, where prior injection into the first eye, should it generate an immune response, may limit expression with later injections of AAV-ND4 into the second eye. LHON is a bilateral disease, thus both eyes need treatment.

Great care must be taken in extrapolating the results achieved in rodents to the human disorder, particularly under pathological conditions. As an example, immunoprecipitation of Complex I following intravitreal injections of the normal allotopic human ND4 revealed a greater distribution of the FLAG-tagged ND4 in the murine optic nerve than that observed in the retina [87]. In contrast, the mutant ND4 had greater incorporation into the murine retina than in the optic nerve [83]. These findings suggest that cellular events associated with the optic disc edema may impede movement of the ND4 integrated into the holoenzyme from the retina to the nerve. The studies of Oca-Cossio suggest that if ND4 is not correctly processed into mitochondria, it may be harmful [91]. In addition, if LHON is primarily an axonopathy, then the allotopic ND4 may not get to the target tissue (axonal mitochondria in the optic nerve) for rescue in acute LHON patients who typically have optic nerve head swelling. On the other hand, if LHON is primarily a disorder of RGCs, then it will rescue. Further studies in lower vertebrates are needed to delineate the best window for intervention.

Many other experimental techniques have been proposed to address disorders caused by mutated mtDNA. They include mitochondrial gene replacement in embryonic stem cells [109], protoFection [110], importing genes from other species, changing the ratio of heteroplasmy with specific restriction endonucleases [111], or selecting for respiratory function or regeneration (in muscle) [112, 113]. None of these techniques are directly applicable to the treatment of LHON that is caused predominantly by 100% mutated mtDNA. An approach worth mentioning here is that of transkingdom allotopic expression coined “xenotopic expression.” This technique was pioneered by Ojaimi and coworkers, who experimentally restored defects in Complex V of the electron transport chain [114]. Using a similar approach, Seo et al. used the NDI1 gene of Saccharomyces cerevisiae to rescue the respiratory deficiency of Complex I deficient Chinese hamster CCL16-B2 cell lines [115]. The NDI1 gene is a single subunit NADH-ubiquinone oxidoreductase that appears to perform the function of the 45 subunit mammalian Complex I. NDI1 is encoded in the nuclear genome, expressed on cytoplasmic ribosomes, and successfully transported into the mitochondrial inner membrane with an N-terminus mitochondrial targeting sequence. 

The Yagi laboratory has applied their NDI1 technology to alleviating the consequences of a human cell line carrying a homoplasmic frame shift mutation in the ND4 gene [116]. Recently, they utilized an AAV expressing NDI1 to rescue the Complex I deficiency induced by rotenone in the mouse visual system [117]. Xenotopic technology has the advantage whereby a single construct, NDI1, can treat all LHON cases caused by mutated ND4, ND1, or ND6 Complex I subunits. Allotopic expression of human Complex I subunits requires three separate constructs, one for each of the three mutated subunit genes. Still, it is unclear whether introduction of a gene from an entirely different species is acceptable for human therapy. Moreover, it has not been demonstrated how NDI1 interacts with Complexes II–V of the human respiratory chain. A recent publication showing the extension of the fly lifespan with the NDI1 gene suggests that the mechanism of benefit was not achieved by improving oxidative phosphorylation, but rather by decreased production of ROS [118]. Still, who is an appropriate candidate for allotopic or xenotopic gene therapy?

## 10. Candidates for Genetic Therapies

The stage of disease may dictate the outcome of gene therapy. Lam and coworkers found that optical coherence tomography (OCT) measurements of the retinal nerve fiber layer (RNFL) averaged 72 *μ*m for as long as 3 years after visual loss [119]. After this time, RNFL thickness dropped to 42 *μ*m. With loss of more than half of their RGCs, these late-stage patients may not have a sufficient population of remaining cells for meaningful rescue of vision. Still, the scAAV that expressed in almost all RGCs of the mouse has the potential to restore function even in those remaining axons described in autopsied LHON eyes as exhibiting accumulation of mitochondria and dissolution of cristae [15]. If so, visual function may improve even with long-standing optic atrophy. That this may be possible is suggested by the Leber's congenital amaurosis (LCA) clinical trials where partial return of visual function occurred even in eyes with severe and long-standing photoreceptor loss [97, 120, 121]. 

The rapidity of gene expression is clinically relevant in treating LHON patients who present with bilateral simultaneous onset of acute visual loss. Still, oxidative injury and apoptosis may already be irreversible at this time. Considering the window period of 2-3 months between the involvement of the first and the second eye, treatment may even be employed before loss of vision. Thus, rescue prior to visual loss in the second eye may be possible during this window period, particularly if introduction of the normal ND4 subunit gene in those eyes with acute optic disc edema after visual loss proves ineffective.

The studies of Elouze et al. suggest that gene therapy prevents visual loss in lower vertebrates [84]. Whether successful rescue in symptomatic patients will support intervention in asymptomatic carriers may be dependent on tests capable of predicting conversion to the phenotype. Using the PERG as a sensitive measure of ganglion cell function, Lam et al. found that the PERG amplitude was substantially reduced in some asymptomatic G11778A carriers [119]. They plan to follow those patients for several years to see if they develop LHON.

In summary, due to the research of many groups all over the world who are working in the field, the molecular and biochemical basis of this disease has been unraveling. Efficient gene delivery techniques tested in vertebrae animal models that mimic the optic nerve degeneration of LHON provide a renewed hope for an effective and long-lasting remedy for this disorder in the coming years.

## Figures and Tables

**Table 1:** Comprehensive list of genes/mutations involved in LHON.

Genes	Gen Bank ID	Nucleotide position	AA change	Phenotype	Hom/Het	Reference
*MT-ND1*	ACT53094.1	m.3316G>A	A4T	LHON/NIDDM	Hom	Matsumoto et al., 1999 [122]
		m.3376G>A	E24K	LHON/MELAS	Hom/Het	Blakely et al., 2005 [123]
		m.3394T>C	Y30H	LHON/NIDDM	Hom	Brown et al., 1992 [124]
		m.3460G>A	A52T	LHON	Hom/Het	Huoponen et al., 1991 [30]
		m.3496G>T	A64S	LHON	Hom	Matsumoto et al., 1999 [122]
		m.3497C>T	A64V	LHON	Hom	Matsumoto et al., 1999 [122]
		m.3635G>A	S110N	LHON	Hom	Brown et al., 2001 [125]
		m.3700G>A	A112T	LHON	Hom	Fauser et al., 2002 [126]
		m.3733G>A	E143K	LHON	Hom/Het	Valentino et al., 2004 [127]
		m.4025C>T	T240M	LHON	Hom	Huoponen et al., 1993 [128]
		m.4136A>G	Y277C	LHON	Hom	Howell et al., 1991 [129]
		m.4160T>C	L286P	LHON	Hom	Howell et al., 1991 [129]
		m.4171C>A	L289M	LHON	Hom/Het	Kim et al., 2002 [130]
		m.4216T>C	Y304H	LHON/Insulin resistance	Hom	Johns and Berman, 1991 [131]

*MT-CO1*	ACT53096.1	m.6261G>A	A120T	LHON/Prostrate Cancer	Hom	Abu-Amero and Bosley et al., 2006 [132]
		m.7444 G>A	Ter-K	LHON/SNH/DEAF	Hom	Brown et al., 1992 [133]
		m.7623 C>T	T13I	LHON	Hom	Abu-Amero and Bosley et al., 2006 [132]

*MT-CO2*	ACT53097.1	m.7868C>T	L95F	LHON	Hom	Yang et al., 2009 [134]

*MT-ND2*	ACT53095.1	m.4640C>A	I57M	LHON	Hom	Brown et al., 2001 [125]
		m.4917A>G	N150D	LHON/AMD/Insulin resistance/NRTI-PN	Hom	Johns and Berman, 1991 [131]
		m.5244G>A	G259S	LHON	Het	Brown et al., 1992 [135]

*MT-ND3*	ACT53101.1	m.10237T>C	I60T	LHON	Hom	Horvath et al., 2002 [136]

*MT-ND4*	ACT53103.1	m.11253T>C	I165T	LHON	Hom	Kjer 1959 [137]
		m.11696G>A	V312I	LHON + Spastic Dystonia	Het	De Vries et al., 1996 [138]
		m.11778G>A	R340H	LHON	Hom/Het	Wallace et al., 1988 [6]
		m.11874C>A	T372N	LHON	Hom	Abu-Amero and Bosley et al., 2006 [132]

*MT-ND4L*	ACT53102.1	m.10543A>G	H25R	LHON	Het	Abu-Amero and Bosley et al., 2006 [132]
		m.10591T>G	F41C	LHON	Het	Abu-Amero and Bosley et al., 2006 [132]
		m.10663T>C	V65A	LHON	Hom	Brown et al., 2002 [139]
		m.10680G>A	A71T	LHON	Hom	Yang et al., 2009 [134]

*MT-ND5*	ACT53104.1	m.12782T>G	I149S	LHON	Het	Abu-Amero and Bosley et al., 2006 [132]
		m.12811T>C	Y159H	LHON	Hom	Huoponen et al., 1993 [128]
		m.12848C>T	A171V	LHON	Het	Mayorov et al., 2005 [140]
		m.13045A>C	M237L	LHON/MELAS/LS	Het	Liolitsa et al., 2003 [141]
		m.13051G>A	G239S	LHON	Hom	Howell et al., 2003 [142]
		m.13379A>C	H348P	LHON	Hom	Abu-Amero and Bosley et al., 2006 [132]
		m.13528A>G	T398A	LHON-Like	Hom	Batandier et al., 2000 [143]
		m.13637A>G	Q434R	LHON	Hom	Huoponen et al., 1993 [128]
		m.13708G>A	A458T	LHON/MS risk	Hom	Johns and Berman, 1991 [131]
		m.13730G>A	G465E	LHON	Het	Howell et al., 1993 [144]
*MT-ND6*	ACT53105.1	m.14568C>T	G36S	LHON	Hom	Besch et al., 1999 [145]
		m.14279G>A	S132L	LHON	Hom	Zhadanov et al., 2005 [146]
		m.14459G>A	A72V	LHON + Spastic Dystonia	Hom/Het	Jun et al., 1994 [147]
		m.14482C>G	M64I	LHON	Hom/Het	Howell et al., 1998 [148]
		m.14484T>C	M64V	LHON	Hom/Het	Brown, et al., 1992 [124]
		m.14495A>G	L60S	LHON	Het	Chinnery et al., 2001 [149]
		m.14498C>T	Y59C	LHON	Hom/Het	Wissinger et al., 1997 [150]
		m.14596A>T	I26M	LHON	Hom	De Vries et al., 1996 [138]
		m.14325T>C	N117D	LHON	Hom	Howell et al., 2003 [142]
		m.14729G>A	S132L	LHON	Hom	Zhadanov et al., 2005 [146]

*MT-CYB*	ACT53106.1	m.14831G>A	A29T	LHON	Hom	Fauser et al., 2002 [126]
		m.14841A>G	N32S	LHON	Het	Yang et al., 2009 [134]
		m.15257G>A	D171N	LHON	Hom	Johns and Berman, 1991 [131]
		m.15674T>C	S310P	LHON	Hom	Abu-Amero Bosley et al., 2006 [132]
		m.15773G>A	V343M	LHON	Hom	La Morgia et al., 2008 [151]
		m.15812G>A	V356M	LHON	Hom	John et al., 1991 [152]

*MT-CO3*	ACT53100.1	m.9438G>A	G78S	LHON	Hom	Johns and Neufeld 1993 [153]
		m.9738G>T	A178S	LHON	Hom	Johns and Neufeld 1993 [153]
		m.9804G>A	A200T	LHON	Het	Johns and Neufeld 1993 [153]

MT-ATP6	ACT53099.1	m.8836A>G	M104V	LHON	Hom	Abu-Amero Bosley et al., 2006 [132]
		m.9016A>G	I164V	LHON	Het	Povalko et al., 2005 [154]
		m.9101 T>C	I192T	LHON	Hom	Puomila et al., 2007 [17]
		m.9139G>A	A205T	LHON	Hom	La Morgia et al., 2008 [151]
